# The Importance of International Collaborations to Advance Research Endeavors

**DOI:** 10.1371/journal.ppat.1006047

**Published:** 2017-01-05

**Authors:** Alfredo G. Torres

**Affiliations:** 1 Department of Microbiology and Immunology, University of Texas Medical Branch, Galveston, Texas, United States of America; 2 Department of Pathology, University of Texas Medical Branch, Galveston, Texas, United States of America; The Fox Chase Cancer Center, UNITED STATES

Growing up in Central Mexico, I was fascinated by the popular belief that the indigenous people used “Montezuma’s revenge” (better known as traveler’s diarrhea) as a way to retaliate against the conquerors that colonized Latin America. As I made the study of diarrheagenic *Escherichia coli* the central pillar of my scientific career, it was evident that the simplistic concept of a vendetta against foreigners was an urban legend. Instead, diarrheal disease results from a complex interplay between different categories (pathovars) of *E*. *coli*, the environment, and the host’s immune response.

As I continued in my study of *E*. *coli*, I became particularly interested in enterohemorrhagic *Escherichia coli* (EHEC). This *E*. *coli* pathovar causes a broad spectrum of disease, ranging from mild diarrhea to hemorrhagic colitis and hemolytic uremic syndrome. In the United States, contamination of beef and vegetables with EHEC serotype O157:H7 has resulted in numerous multistate outbreaks of diarrheal disease. Our work at the University of Texas Medical Branch has contributed to the growing body of literature regarding EHEC O157:H7 pathogenesis, virulence, and immune evasion. Such knowledge has been translated into the development of vaccines and therapeutics, resulting in reduced EHEC O157:H7-associated disease in humans.

As I witnessed the translation of our research findings to clinical outcomes, my attention became refocused on Latin America. It is well understood that finding a solution for *E*. *coli*-derived diarrheal disease and associated complications in this region would require a multidisciplinary team approach. Latin American investigators have a long-standing tradition of contributing to important advances in the discovery and characterization of different *E*. *coli* pathovars. For this reason, it was important to not only improve the visibility of their work but also to establish the collaborative network necessary to identify and implement solutions in their countries.

During informal discussions with *E*. *coli* experts in the Latin American region, it became apparent that globalization of the food industry and its impact on agricultural trade have resulted in increased dissemination of pathogenic *E*. *coli* strains to different countries. Furthermore, since each country possesses their own autochthonous *E*. *coli* pathovars, the emergence of new outbreak-associated *E*. *coli* strains poses a serious concern. Such strains could have a devastating impact on animal and agricultural trade, as well as human health.

It was clear that a coordinated, multidisciplinary approach would be required in order to identify, diagnose, characterize, and further understand these pathogenic *E*. *coli* strains and their associated diseases. To address this need for a collaborative effort, the Latin American Coalition for *E*. *coli* Research (LACER) was formed in 2009. LACER is comprised of a diverse group of individuals dedicated to promoting and expanding *E*. *coli* research projects, with particular focus on training and supporting the next generation of Latin American researchers. This coalition represents a multidisciplinary group of bacteriologists, public health officials, epidemiologists, molecular biologists, veterinarians, physicians, and other health-related experts distributed across more than 60 research groups located in different Latin American countries, Canada, and the United States.

What is unique about LACER that makes it a successful collaborative group of researchers? First, there is no fee to become a member. The only requirement is a full commitment to collaborate with other groups, both nationally and internationally, in an effort to advance pathogenic *E*. *coli* research. Second, LACER does not have a complex leadership team; instead, members are frequently consulted and participate in the decision-making process of all activities planned. Third, LACER members are devoted to improving the quality of education by sharing resources and allowing other groups to have access to equipment, reagents, and protocols that they might not have in their own laboratory. Finally, members can communicate their research findings through various symposiums, colloquiums, mini-courses, and social media outlets coordinated by LACER. Additionally, members are encouraged to increase the number and quality of their publications via an internal peer-review process.

Significant contributions have emerged from this international collaborative coalition, including five LACER symposiums, with more than 400 research posters presented by trainees since 2009. Importantly, the publication rate of *E*. *coli* research has been steadily increasing since the inception of LACER, with approximately 15% of the publications corresponding to collaborative research efforts between the LACER members. Additionally, by publishing two books, LACER continues to contribute to the education of the new generation of *E*. *coli* researchers. The most recent book, *Escherichia coli in the Americas*, covers basic concepts regarding different *E*. *coli* pathovars, including their environmental niche, virulence mechanisms, and host reservoir. This book also addresses disease outcomes, diagnosis, treatment, and vaccine development. However, perhaps the most important contribution of LACER has been the recent observation that “hybrid” *E*. *coli* pathovars (signature virulence factors from one pathovar found in another *E*. *coli* category) are currently emerging in Latin America and causing human disease. The potential of these strains to cause outbreaks has encouraged LACER members to become more vigilant and increase their exchange of information in order to detect and prevent the emergence of such strains.

As founder and current coordinator of LACER, my goal has been to facilitate interactions between groups of committed investigators in order to make the legends of “Montezuma’s revenge,” the “Aztec curse,” the “Delhi belly,” and “Gyppy tummy” a thing of the past. Through these effective collaborative research efforts, we can directly impact the well-being of these Latin American communities and the rest of the world.

**Image 1:**
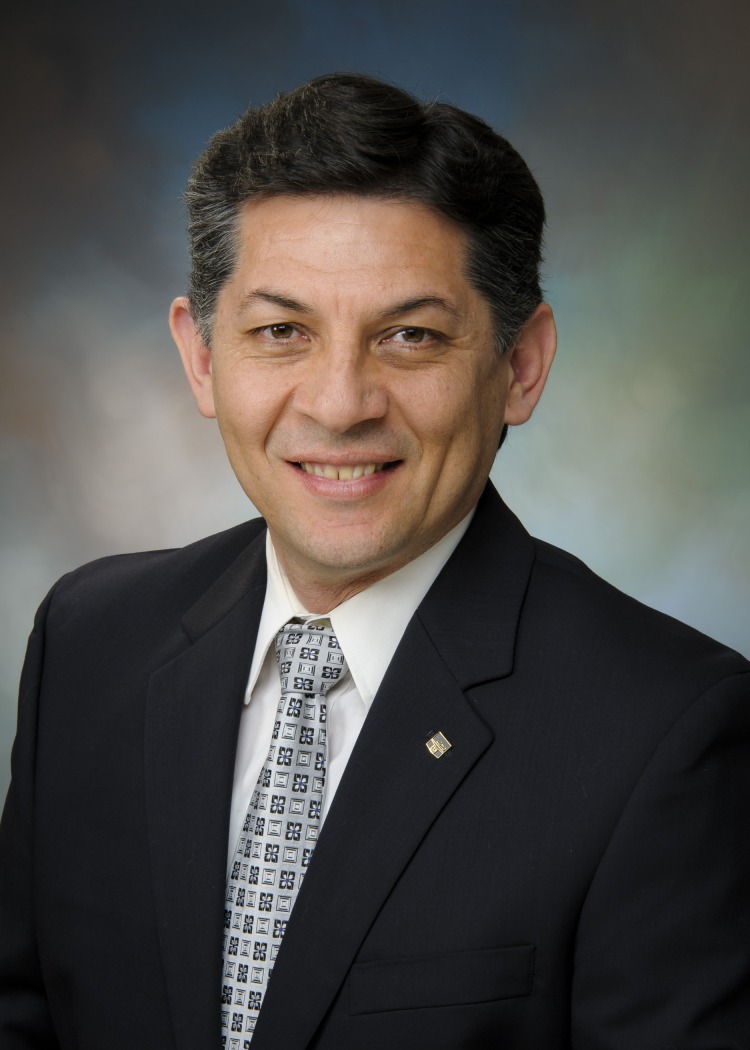
Alfredo G. Torres.

